# Emerging modes of PINK1 signaling: another task for MARK2

**DOI:** 10.3389/fnmol.2014.00037

**Published:** 2014-05-08

**Authors:** Dorthe Matenia, Eva M. Mandelkow

**Affiliations:** ^1^Max-Planck-Institute for Neurological ResearchHamburg, Germany; ^2^German Center for Neurodegenerative Diseases–Center of Advanced European Studies and ResearchBonn, Germany

**Keywords:** PINK1, MARK2, mitochondria, transport, differentiation, neurodegeneration, Alzheimer disease, Parkinson disease

## Abstract

PTEN-induced kinase 1 (PINK1) acts at multiple levels to promote mitochondrial health, including regulatory influence on ATP-synthesis, protein quality control, apoptosis, mitochondrial transport, and destiny. PINK1 mutations are linked to Parkinson disease (PD) and mostly result in loss of kinase activity. But the molecular events responsible for neuronal death as well as the physiological targets and regulators of PINK1 are still a matter of debate. This review highlights the recent progress evolving the cellular functions of the cytosolic pool of PINK1 in mitochondrial trafficking and neuronal differentiation. Regulation of PINK1 signaling occurs by mitochondrial processing to truncated forms of PINK1, differentially targeted to several subcellular compartments. The first identified activating kinase of PINK1 is MAP/microtubule affinity regulating kinase 2 (MARK2), which phosphorylates T313, a frequent mutation site linked to PD. Kinases of the MARK2 family perform diverse functions in neuronal polarity, transport, migration, and neurodegeneration such as Alzheimer disease (AD). This new protein kinase signaling axis might provide a link between neurodegenerative processes in AD and PD diseases and opens novel possibilities in targeting pathological signaling processes.

## INTRODUCTION

Many neurodegenerative disorders, such as Alzheimer (AD) and Parkinson disease (PD) show mitochondrial abnormalities during their pathogenesis. Neurons, due to their size and complex geometry, are particularly dependent on the proper functioning and distribution of mitochondria, which are the powerhouses of the cells. Beside ATP production, they perform a variety of functions that are important for cell life and death, including reactive oxygen species (ROS) generation, intracellular calcium homeostasis, and apoptosis. In the nervous system, mitochondrial dynamics are crucial to guarantee long distance delivery and balanced distribution of energy to axons, dendrites and synapses (Jacobson and Duchen, 2004; DiMauro and Schon, 2008). Tau and other microtubule associated proteins promote the assembly and stabilization of neuronal microtubule tracks and ensure microtubule dependent transport. Pathological changes of tau may lead to the breakdown of microtubules observed in AD while elevated tau on microtubules can compete with motor proteins, resulting in inhibition of traffic (Mandelkow et al., 2004; Dixit et al., 2008). This suggests that a strict regulation is needed to maintain the flow of material. Phosphorylation of tau, especially at the KXGS motifs of the repeat domain, decreases its affinity to microtubules and provides a mechanism for regulating microtubule stability as well as axonal transport (Matenia and Mandelkow, 2009). Enhanced phosphorylation of tau at multiple sites is an early hallmark of AD, followed by abnormal aggregation of tau protein into paired helical filaments (PHFs) and neurofibrillary tangles (NFTs). The microtubule-affinity regulating kinase 2 (MARK2) was originally discovered by its ability to phosphorylate tau protein and related microtubule-associated proteins (MAPs; Drewes et al., 1997; Schwalbe et al., 2013). Furthermore, active MARK2 co-localizes with NFTs in AD brain, and MARK2 target sites on tau are elevated in transgenic mouse models of tauopathy, emphasizing the importance of MARK2 in this disease (Matenia and Mandelkow, 2009). Recently, MARK2 was identified as an upstream regulator of PTEN-induced kinase 1 (PINK1; Matenia et al., 2012). This provides insights into the regulation of mitochondrial trafficking in neurons and a potential link between neurodegenerative processes in AD and PD.

## PTEN-INDUCED KINASE 1

Familial cases of PD can be caused by mutations in different genes, such as PINK1 or Parkin. PINK1 is a mitochondria-targeted serine/threonine kinase promoting cell survival, particularly under conditions of oxidative/metabolic stress (Valente et al., 2004; Deng et al., 2005; Wood-Kaczmar et al., 2008). In particular, PINK1 regulates mitochondrial transport, morphology, biogenesis, function, calcium buffering capacity, and mitochondrial clearance (Petit et al., 2005; Wood-Kaczmar et al., 2008; Dagda and Chu, 2009; Gandhi et al., 2009; Gegg et al., 2009; Van Laar and Berman, 2009; Matsuda et al., 2010; Narendra et al., 2010; Sun et al., 2012). Most of the reported PD-linked PINK1 mutations result in a loss of kinase activity (Cookson and Bandmann, 2010).

**FIGURE 1:**
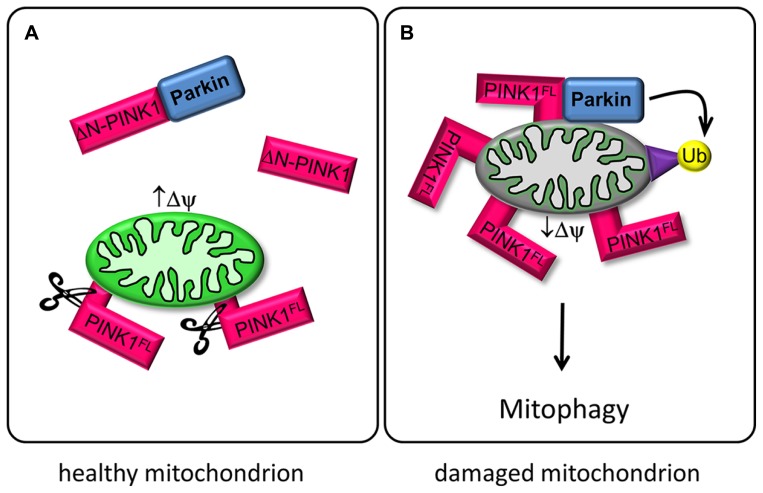
**PINK1 and Parkin regulate mitophagy. (A)** In healthy mitochondria with high mitochondrial membrane potential (↑ΔΨ), PINK1^FL^ is maintained at low levels by the sequential proteolytic actions of mitochondrial processing peptidase (MPP) and presenilin-associated rhomboid-like protease (PARL; Greene et al., 2012). The resulting ΔN-PINK1 is partially located in the cytosol and interacts directly with Parkin, thereby preventing Parkin-mediated mitophagy (Fedorowicz et al., 2014). **(B)** Upon mitochondrial depolarization (↓ΔΨ) PINK1^FL^ is stabilized and selectively accumulates in the outer membrane of defective mitochondria with its kinase domain facing the cytoplasm. This accumulation is a crucial signal for Parkin recruitment to impaired mitochondria, promoting ubiquitination of mitochondrial outer membrane proteins and subsequent disposal of the damaged organelle (Narendra et al., 2010).

The molecular events responsible for PINK1-induced neuronal death as well as its physiological substrates or regulators are still a matter of debate (Deas et al., 2009; Pogson et al., 2011). Upon entry to the mitochondria the PINK1 protein is proteolytically cleaved by mitochondrial processing peptidase (MPP) and presenilin-associated rhomboid-like protease (PARL) to produce two N-terminally truncated protein fragments of 54 and 45 kDa without mitochondrial localization sequence (Deas et al., 2009; Narendra et al., 2010; Greene et al., 2012). The cleaved ΔN-PINK1 forms localize preferentially in the cytosolic instead of the mitochondrial fraction (Lin and Kang, 2008). ΔN-PINK1 is constitutively degraded in the cytosol by the proteasomal pathway (Yamano and Youle, 2013), indicating that only the mitochondrially targeted PINK1^FL^ has a cellular function. But expression of ΔN-PINK1 protects neurons against the neurotoxin 1-methyl-4-pheny-1,2,3,6-tertahydropyridin (MPTP). This suggests that the mitochondrial import sequence of PINK1 is not strictly necessary for neuroprotection and that cytosolic targets and signal transduction pathways may be modified by cleaved PINK1 (ΔN-PINK1) to affect neuronal survival (Haque et al., 2008). Recent studies validate this hypothesis. PINK1 cleavage-products localized in the cytosol are degraded by proteasomes but also bind Parkin, repress Parkin translocation to mitochondria and prevent mitophagy (Fedorowicz et al., 2014; **Figure 1A**). Furthermore, cytosolic ΔN-PINK1 influences mitochondrial mobility. The kinase enhances anterograde movements of mitochondria, both in dendrites and axons (Matenia et al., 2012; Dagda et al., 2013). However, the mechanisms of these ΔN-PINK1 functions are mostly unknown. So far only one upstream regulating kinase was identified: MARK2 phosphorylates PINK1 and thereby regulates mitochondrial transport parameters (Matenia et al., 2012). This new signaling axis might help to clarify common mechanisms in neurodegenerative diseases, although future studies are required to understand the exact functional relationship of these kinases.

## REGULATION OF PINK1 AND MITOCHONDRIAL MOTILITY IN NEURONS

Recent studies have investigated the PINK1/Parkin pathway for sensing and selectively eliminating damaged mitochondria from the mitochondrial network. Parkin is a cytoplasmic E3 ubiquitin ligase and can be phosphorylated by PINK1 (Kim et al., 2008). Both proteins cooperate to control mitochondrial clearance, known as mitophagy. Full length PINK1 (PINK1^FL^) is stabilized on mitochondria with low membrane potential and recruits cytosolic Parkin, which becomes enzymatically active and initiates the lysosomal degeneration of defective mitochondria via ubiquitination of mitochondrial target proteins (Youle and Narendra, 2011; Grenier et al., 2013; **Figure 1B**).

Another aspect of PINK1 concerns its role in the regulation of mitochondrial transport in neurons (Wang et al., 2011; Liu et al., 2012; Matenia et al., 2012; Dagda et al., 2013). Mitochondria are transported along microtubules by the motor proteins kinesin (anterograde, toward the microtubule plus ends) and dynein (retrograde). The kinesin-adaptor complex attached to the outer mitochondrial membrane comprises the GTPase Miro, kinesin heavy chain, and the adaptor protein Milton (Goldstein et al., 2008). PINK1^FL^ is also attached to this complex, and even ΔN-PINK1 can be targeted to it despite the lack of the mitochondrial targeting sequence (Weihofen et al., 2009). PINK1^FL^ phosphorylates the GTPase Miro, and thus induces Parkin-dependent degeneration of Miro. The resulting decrease in mitochondrial movement may represent a quality control mechanism of defective mitochondria (Wang et al., 2011). Active mitochondria with a high membrane potential tend to cause cleavage of PINK1^FL^ to ΔN-PINK1, which is released to the cytoplasm, where it is not only destined for degradation by the proteasome but also binds to Parkin (Miller and Sheetz, 2004; **Figure 1A**). The interaction of cytosolic ΔN-PINK1 with Parkin represses Parkin translocation to the mitochondria and subsequent mitophagy (Fedorowicz et al., 2014). The question is therefore how the different isoforms of PINK1 become active.

Only few studies have examined the regulation of PINK1 and its consequences for mitochondria. The serine/threonine kinase MARK2 phosphorylates PINK1, activates the kinase activity of ΔN-PINK1 (with regard to the artificial substrate histone H4) and enhances protein stability of both, PINK1^FL^ and ΔN-PINK1, arguing for a physiological relevance of this kinase-substrate interaction. The primary phosphorylation site is threonine 313 (T313; Matenia et al., 2012). This residue is mutated to a non-phosphorylatable form (T313M) in a frequent variant of PD (Mills et al., 2008). Residue T313 is located in β-strand 5 of PINK1 (based on a structural model of PINK1 by Swiss-Model using CaMK1 as a structure template; Matenia et al., 2012). Phosphorylation of this residue could therefore result in the interaction of this strand with helix C which is part of the scaffold that fixes the Mg-ATP underneath the P-loop. Stabilization of this part of the catalytic domain is a requirement for the activity of the kinase. In fact, mutation of T313 to glutamate further enhances the phosphorylation and activation of ΔN-PINK1 by MARK2, suggesting that this residue could be a priming phosphorylation site, changing the conformation of the kinase and preparing it for further modifications.

**FIGURE 2:**
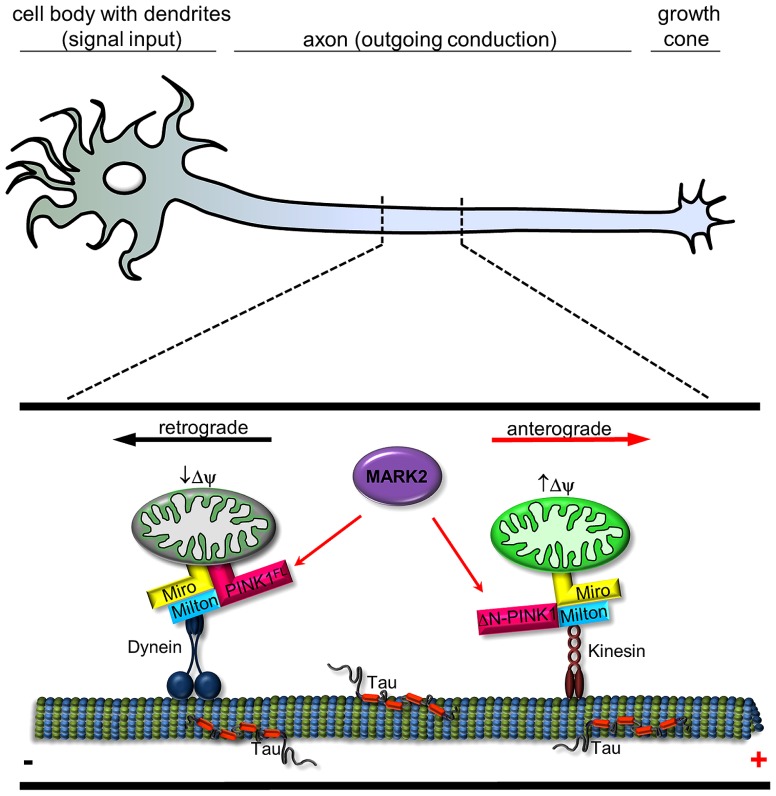
**Schematic representation of interplay between MARK2 and PINK1^FL^/ΔN-PINK1 to regulate mitochondrial transport.** In a healthy neuron, mitochondria are carried along by motor proteins dynein (retrograde) and kinesin (anterograde). PINK1 is a molecular switch that changes the probability between anterograde and retrograde mitochondrial transport. Transport direction of neuronal mitochondria is regulated by PINK1 cleavage and binding/phosphorylation by MARK2. Kinesin motors are linked to mitochondria by adaptor proteins like Miro and Milton (Weihofen et al., 2009) and regulate in association with ΔN-PINK1 the anterograde movement (*red arrow*; ), whereas Miro also has an effect on dynein-mediated retrograde movement (Russo et al., 2009; *black arrow*). Active mitochondria tend to cause cleavage of PINK1^FL^ (Narendra et al., 2010), which is phosphorylated at Thr-313 by MARK2 (Matenia et al., 2012); both events promote anterograde movement by kinesin. Retrograde movement by dynein is promoted by PINK1^FL^ and further increased by MARK2 (Matenia et al., 2012).

The importance of the PINK1 phosphorylation site T313 is further emphasized by the fact that expression of PINK1^T313M^ causes severe toxicity for cells. ΔN-PINK1^T313M^ leads to abnormal mitochondrial accumulation in the cell soma, whereas PINK1^FL/T313M^ causes degradation of mitochondria. Within neurons endogenous PINK1 and MARK2 colocalize partly on mitochondria, especially in axons and dendrites, changing mitochondrial transport parameters (mitochondrial density and movement direction in axons). MARK2 interacts with and preferentially phosphorylates the cytosolic ΔN-PINK1, thereby increasing its kinase activity and promoting anterograde mitochondrial motility (Matenia et al., 2012). Consistent with this, a high membrane potential enhances the anterograde transport of mitochondria (Miller and Sheetz, 2004) and also promotes the proteolysis of PINK1^FL^ into ΔN-PINK1 (Narendra et al., 2010), thereby inhibiting Parkin-dependent mitophagy of active mitochondria (**Figure 2**). Phosphorylation and activation of ΔN-PINK1 by MARK2 possibly enhances the stability of the mitochondrial transport-complex and ensures the supply of energy at the growth cone. Conversely, retrograde transport is favored for mitochondria with low membrane potential destined for mitophagy (Jin et al., 2010). In this case the cleavage of PINK1^FL^ is inhibited. Thus, Parkin decorated mitochondria assemble as large clusters primarily in the lysosome-rich perinuclear area (Narendra et al., 2010). This effect is influenced by MARK2 (Matenia et al., 2012). MARK2 could phosphorylate PINK1^FL^, consequently enhance the binding and possibly the phosphorylation of Parkin and Miro by PINK1^FL^. This results in accumulation of mitochondria around the perinuclear region and suggests that failure of the MARK2-PINK1 signaling cascade could contribute to PD. Thus, our study revealed the existence of two cellular pools of PINK1 that differently modify and regulate mitochondrial movement direction.

## PINK1, MARK2, AND DIFFERENTIATION

MAP/microtubule affinity regulating kinase 2 is involved in several regulatory processes of the cell such as determination of polarity, cell cycle control, intracellular signal transduction, transport, and cytoskeletal stability (Matenia and Mandelkow, 2009). MARK2 and its homolog par-1 (for “partition defective”) belongs to a set of conserved proteins in *Drosophila* and *Caenorhabditis elegans*, which are essential for cellular polarity, with roles in establishing the embryonic body axis and in maintaining cell differentiation (Kemphues et al., 1988; Tomancak et al., 2000). The par-1-dependent cell polarization is based on a tight network of cross-reactive and feedback interactions of the par proteins, other regulators of polarity and the cytoskeleton (Munro, 2006). MARK/par-1 is a central player in localization of the cell polarity proteins. In mammalian epithelial cells the overexpression of inactive MARK2 disturbs the polarity, suggesting a similar mechanism of governing polarization (Böhm et al., 1997).

Microtubules are important determinants of cell polarity. MARK2 plays a significant role in axon formation, which requires dynamic instability of microtubules (Biernat et al., 2002). This is in part related to the phosphorylation of axonal Tau protein in its “repeat domain” which decreases its affinity for microtubules. The reduction of MARK2 via RNA interference (RNAi) induces multiple axons in hippocampal neurons, whereas enhanced MARK2 expression inhibits axon formation altogether (Chen et al., 2006). Following the establishment of an axon MARK2 promotes its elongation (Uboha et al., 2007). In dendrites, the predominant MAP is MAP2 which has a similar repeat domain as Tau and can also be phosphorylated by MARK2 (Illenberger et al., 1996). In this case MARK2 inhibits the development of dendrites in hippocampal neurons through phosphorylation of MAP2. In particular, MARK2 shortens the length and decreases branching of dendrites (Terabayashi et al., 2007).

Interestingly, transient expression of ΔN-PINK1 promotes dendritic outgrowth and neurite length in dopaminergic midbrain neurons. This effect seems to be kinase dependent, since a kinase deficient mutant of PINK1 fails to influence neurite length. The action of ΔN-PINK1 on neurite length was not related to its activity at mitochondria, since an outer mitochondria membrane (OMM)-targeted ΔN-PINK1 construct, which exhibits cytosolic localization, failed to enhance neuronal differentiation. These data indicate divergent roles for cytosolic and mitochondrial targeted forms of PINK1. Furthermore, PINK1 deficiency reduces dendritic length of primary neurons isolated from PINK1 knockout mice. To clarify the mechanism underlying the regulation of neurite outgrowth induced by cytosolic ΔN-PINK1, Dagda et al. (2013) examined the expression of various neuronal differentiation proteins as a function of PINK1. PINK1 increases levels of MAP2 and activates protein kinase A (PKA)-regulated signaling pathways. Since MAP2 is an anchoring protein of PKA in dendrites (Obar et al., 1989; Harada et al., 2002), this data suggests that PINK1 is an upstream regulatory kinase of this pathway to influence dendritic morphology. On the other hand, the ability of microtubule-associated PKA to promote elongation of dendrites is independent of MAP2 phosphorylation. This suggests other proteins in close proximity to the microtubule cytoskeleton are involved in this process (Huang et al., 2013). Since KXGS is not only a kinase consensus motif for targets of MARK2 but also of PKA, both kinases share some substrate preferences (Drewes et al., 1997). The microtubule binding affinity of tau as well as that of doublecortin (Dcx) is regulated via phosphorylation by MARK2 and PKA (Drewes et al., 1997; Schaar et al., 2004). This provides the clue, that MARK2 signaling pathways could be involved in ΔN-PINK1 mediated neurite outgrowth regulation. Due to substrate competition ΔN-PINK1 could inhibit MARK2 by binding, thereby enhancing dendritic length.

## CONCLUSION AND OUTLOOK

This review summarizes and evaluates recent findings in PINK1 biology and focuses on emerging aspects concerning the novel role of cytosolic ΔN-PINK1 that has not yet received adequate attention as compared to mitochondrial PINK1^FL^. In the case of mitochondria the full-length PINK1 regulates the transport and clearance of defective mitochondria through phosphorylation of Miro and recruitment of Parkin, respectively (**Figure 1**). These protective activities of PINK1^FL^ are dependent on its localization at the mitochondrial surface and have been studied extensively. But even the N-terminally truncated enzyme ΔN-PINK1 lacking the mitochondrial localization signal can be found in close proximity to mitochondria, probably via binding to mitochondrial membrane localized protein adaptor complexes (**Figure 2**), controlling their health status and distribution. Beside this task, ΔN-PINK1 released from mitochondria via proteolytic cleavage by mitochondrial enzymes shows neurite promoting activity. This outgrowth effect was specific to dendrites as axonal length did not change significantly (Tieu and Xia, 2013).

Microtubule-affinity regulating kinase 2, the upstream regulator of ΔN-PINK1 and PINK-1^FL^, activates and regulates a diverse range of cellular activities and participates in several signaling cascades. Since the discovery of MARK2 as a kinase of Tau and MAP2 (Drewes et al., 1997), several other substrates have been identified. Some of these affect mitochondrial transport, presumably by regulating the affinity to microtubules (Thies and Mandelkow, 2007; Pluciñska et al., 2012). Neurons are particularly dependent on mitochondrial function, so disrupting the transport of these organelles can cause neurological disease (Schon and Przedborski, 2011). The vulnerability results from the high metabolic demands of neurons, their dependence on proper calcium handling and their susceptibility to local ROS signaling, processes in which mitochondria are critically involved. In the context of transport PINK1 acts as a molecular switch between anterograde and retrograde mitochondrial transport. As mentioned above, transport direction is regulated by PINK1 cleavage depending on the mitochondrial membrane potential (**Figure 1**) and PINK1 binding/phosphorylation by MARK2 (**Figure 2**; Matenia et al., 2012). Since MARK2 is an upstream regulatory component in PINK1 signaling, this extends the complexity of its biological function. Mitochondria are enriched at synapses and play a critical role in both pre- and post-synaptic functions (Hollenbeck, 2005). Accurate regulation of mitochondrial motility and maintenance of neuronal plasticity are closely related.

Increasing evidence implicates that dysfunction of kinase activities and phosphorylation pathways are involved in the pathogenesis of neurodegenerative diseases. PINK1 mutations linked to PD are mostly accompanied by loss of kinase activity; therefore an effective therapy would have to replace functional PINK1-signaling. The limiting factor is that the details of the PINK1 signaling network are not yet fully elucidated. An initial step in the right direction is the identification and characterization of a PINK1/Parkin independent mitophagy pathway (Allen et al., 2013). Selective induction of mitophagy could prove beneficial as a potential therapy for several neurodegenerative diseases in which mitochondrial clearance is advantageous.

## Conflict of Interest Statement

The authors declare that the research was conducted in the absence of any commercial or financial relationships that could be construed as a potential conflict of interest.
